# Reassurance: Medically Unexplained Physical Symptoms

**DOI:** 10.1371/journal.pmed.0030541

**Published:** 2006-12-26

**Authors:** Bill Anderson

Escobar et al.'s discussion of medically unexplained physical symptoms is useful, and could trigger a renewal of how the medical profession works [1]. We are in transition at the present, and what we have been trained for is less and less relevant. This disjunction between our training and practise has given rise to a generation of unhappy doctors.

The traditional “doctor” dealt with symptoms and illnesses for which there was no useful explanation and became expert in managing these. We have completed a century or more of science and pathology and have trained doctors to derive their interventions from an (often cursory) consideration of the patient's illness and the application of reason within this scientific framework, in the unshakable belief that this strategy gave the best and at times the only possibility of assisting the patient in a meaningful way. The practical effects of such practices were rarely if ever examined. This approach had many successes but lots of failures, and we have moved on. We now have built upon the science a growing evidence base which to date principally addresses disease states (pathological processes) rather than illnesses (patients' experiences).

There is however no reason why evidence-based approaches cannot be applied to the management of symptoms and illness as well as disease states. As we develop that evidence base, the nature of clinicians can change. We will not require all “doctors,” for want of a better word, to be comprehensively trained in bioscience. There will be more need for them to deploy skills in implementing well-developed, evidence-based interventions and fitting them to the illness the patient is experiencing. Such “doctors” will deliver most care and pay better attention to the patient's symptoms and illness than we do today. We will of course require another different sort of “doctor” who will do the research and the sifting to develop the evidence base upon which therapies will become universally dependent.
